# Estimating the rate of acute adverse reactions to non-ionic low-osmolar contrast media: a systematic review and meta-analysis

**DOI:** 10.1007/s00330-025-11526-z

**Published:** 2025-04-11

**Authors:** Yuguo Wei, Xinchao Jiang, Mark Hibberd, Alexis Sampedro, Jeannette Rautenbach

**Affiliations:** 1 Medical Affairs, GE Healthcare, Bejing, China; 2 Global Medical Services, GE Healthcare, Marlborough, USA; 3 Medical Affairs, GE Healthcare, Madrid, Spain; 4 Global Medical Affairs, GE Healthcare, Braunschweig, Germany

**Keywords:** Contrast media, Non-ionic, Low-osmolar, Hypersensitivity, Adverse reaction

## Abstract

**Objectives:**

This systematic review and meta-analysis aimed to assess and compare acute adverse reactions (AAR) rates among non-ionic low-osmolar contrast media (LOCM), examining administration routes and severity-specific impact on AAR rates.

**Materials and methods:**

A PubMed and Cochrane Library search identified studies published between January 1989 and March 2024. Inclusion criteria focused on studies with > 100 adult patients who received intra-arterial or intravenous LOCM (iobitridol, iohexol, iomeprol, iopamidol, iopromide, and ioversol). Duplicate reports and studies with insufficient information were excluded. Data extraction and quality assessment followed PRISMA guidelines and the Newcastle Ottawa Scale. Statistical analyses were performed using R software, including random effects, meta-regression, and sub-group analysis.

**Results:**

After excluding duplicates and non-compliant studies, 32 peer-reviewed articles of initially 6701 identified studies, were included in the final analysis. The pooled overall AAR rate was 0.73%, with ioversol showing the lowest rate (0.34%). From all studies, pooled rates (random effects model) of moderate and severe AARs were 0.10% and 0.014% (*p* < 0.01), with the lowest rates for iohexol (0.05% and 0.008%, respectively). The highest overall, moderate, and severe AAR rates were seen with iomeprol (1.38%, 0.27%, and 0.040%, respectively). LOCM type (*p* < 0.0001), study design (*p* = 0.0001), and injection route (*p* = 0.034) significantly influenced the overall AAR rate. In contrast, the study center number (*p* = 0.698), the country where the study was performed (*p* = 0.808), and the type of reaction (hypersensitivity vs hypersensitivity plus physiological reactions; *p* = 0.178) did not.

**Conclusion:**

AAR rates were low but indicated significant differences between LOCM; iohexol and ioversol demonstrated the overall most favorable safety profiles.

**Key Points:**

***Question*** *Knowledge about AAR is crucial for patient safety, but comprehensive data on the safety profiles of non-ionic LOCM is lacking*.

***Findings*** *Ioversol showed the lowest overall AAR rate; iohexol demonstrated the lowest moderate/severe AAR. Study design, LOCM type, and injection route influenced AAR rates*.

***Clinical relevance*** *This meta-analysis provides evidence for differences in non-ionic LOCM safety profiles, particularly for moderate and severe AARs. These can guide clinicians in selecting contrast agents, aiming to further reduce risks, and improve patient safety in diagnostic imaging*.

## Introduction

Contrast media are indispensable in providing relevant clinical information during various radiological procedures [1]. Iodinated contrast media (ICM) are primarily used for imaging vascular conditions, tumors, and other organ abnormalities [2]. They are classified into ionic and non-ionic agents, with further categorization based on osmolality: high-osmolar contrast media (HOCM), low-osmolar contrast media (LOCM), and iso-osmolar contrast media (IOCM). HOCM has an osmolality 5–8 times higher than blood, LOCM 2–3 times higher than blood, depending on concentration, and IOCM has an osmolality similar to that of blood at all concentrations [3]. Due to patient safety concerns, LOCM and IOCM are nowadays the predominantly used ICMs for intravascular administrations in X-ray imaging procedures.

Despite these developments, ICM still poses a small risk of acute adverse reactions (AARs), with reported prevalences varying, ranging from 0.77% to 1.74% according to a recent meta-analysis [4]. Most AARs are mild hypersensitivity (allergic-like) reactions (HSR) such as erythema or urticaria, or physiological reactions like nausea and vomiting. Moderate AARs including protracted nausea or vomiting, diffuse erythema, or facial edema without dyspnea, are less common (0.2–0.4% for LOCM/IOCM). They are more pronounced and often require medical attention. Although LOCM and IOCM are associated with a lower risk of AARs than HOCM, severe reactions such as pulmonary edema, cardiac arrhythmias, anaphylaxis, and seizures may occur (0.04%) and may be life-threatening, requiring immediate medical attention [2, 5–7]. Severe AARs may require follow-up interventions or further hospitalization, and they remain a significant clinical challenge and threat to patients and clinicians. This systematic review and meta-analysis aimed to assess the AAR rates of LOCM, evaluate differences in AAR rates between LOCM types and routes of administration, and analyze severity-specific differences in AAR rates between LOCM types.

## Materials and methods

### Databases and search terms

Electronic databases such as PubMed and Cochrane Library were searched to identify articles published between January 1989 and March 2024. The systematic literature search, along with data selection and extraction, was conducted and reported using the preferred reporting items for systematic reviews and meta-analyses (PRISMA) guidelines [8]. Medical subject heading search terms, such as “acute adverse reaction, adverse reaction, allergy, anaphylaxis, complications, hypersensitivity, iobitridol, ICM, iohexol, iomeprol, Iomeron, iopamidol, Iopamiro, iopromide, ioversol, Isovue, non-ionic, Omnipaque, Optiray, safety, side effect, tolerability, toxicity, Ultravist, Xenetix” were used. They were separated by suitable Boolean operators to identify relevant articles for comparing six LOCM (iobitridol, iohexol, iomeprol, iopamidol, iopromide, and ioversol). The search excluded ioxaglate, an ionic LOCM mostly used in interventional cardiology, and the IOCM iodixanol to limit comparison to only one class of ICM, i.e., non-ionic LOCM.

### Search strategy

Articles published in English were searched and duplicates were removed after manual curation. Identified publications were then screened by title and abstract, excluding meeting abstracts, conference abstracts with insufficient data, case report reviews, and meta-analyses. Only full-text articles meeting the selection criteria were included in the meta-analysis.

### Study selection

All articles were independently screened and reviewed by two authors, Y.W. and X.J., with experience in meta-analysis and medicine. Inclusion criteria for eligibility were: (i) studies investigating the rate of AARs to at least one of the six LOCM specified in the search terms, (ii) study populations comprising over 100 adults (aged > 18 years) from a general population for each type of LOCM, (iii) patients receiving either intra-arterial (IA) or intravenous (IV) ICM and (iv) studies providing sufficiently detailed descriptions of data to enable the extraction of AAR rates (HSR and/or physiological reactions) to particular LOCM within 1 h of administration. Duplicate reports, animal studies, studies with insufficient information, or studies based on a previously published study were not considered.

### Data extraction

The extracted data included study characteristics (authors, publication year, study period, country, study design, and study site), demographic characteristics (age and sex), and results detailing the reported number of overall, moderate, and severe AARs for each type of LOCM.

### Quality assessment

The quality of the studies included in this systematic review was assessed using the Newcastle Ottawa Scale for quality assessment of cohort studies, a tool to evaluate the quality of non-randomized studies in meta-analyses [9]. The scoring system ranged from 0 to 9, with higher scores indicating better study quality. Study characteristics and level of evidence were recorded. Missing data points were carefully evaluated to ensure data quality.

### Statistical analysis

The statistical software R (version 4.1.3) was used to perform the statistical analysis. The overall AAR rate for each study was calculated by dividing the number of patients who experienced an AAR by the total number of patients. For each LOCM type, the overall AAR rate was estimated using the software’s ‘meta’ and ‘metafor’ packages, which employ logistic regression to the dataset, incorporating a random effects model to account for variations in true effect sizes between studies.

Statistical heterogeneity between studies, potentially arising from differences in populations, interventions, outcomes, or methods, was assessed using *I*² statistics and heterogeneity variance τ². To investigate and assess its causes, subgroup analyses of study characteristics that may have influenced outcomes were performed, including study design, route of LOCM administration (IA/IV), single/multi-center studies, and HSR or HSR plus physiological reactions. Mixed-effects (univariate and multivariate) meta-regression analyses were conducted to assess the effect of LOCM type on AAR rates. Funnel plots were used to evaluate potential publication bias. Each point on the plot represents a study (*x*-axis showing effect size, *y*-axis showing measure of standard error). In the absence of bias, the plot should resemble a symmetrical inverted funnel. The ‘dmetar’ package in R was used to perform a sensitivity analysis, which can analyze the influence using the leave-one-out method. In the forest plots, the recalculated pooled proportions and the *I*^2^ values are reflected omitting one study at a time.

## Results

### Baseline characteristics

#### Studies identified

**Fig. 1:**
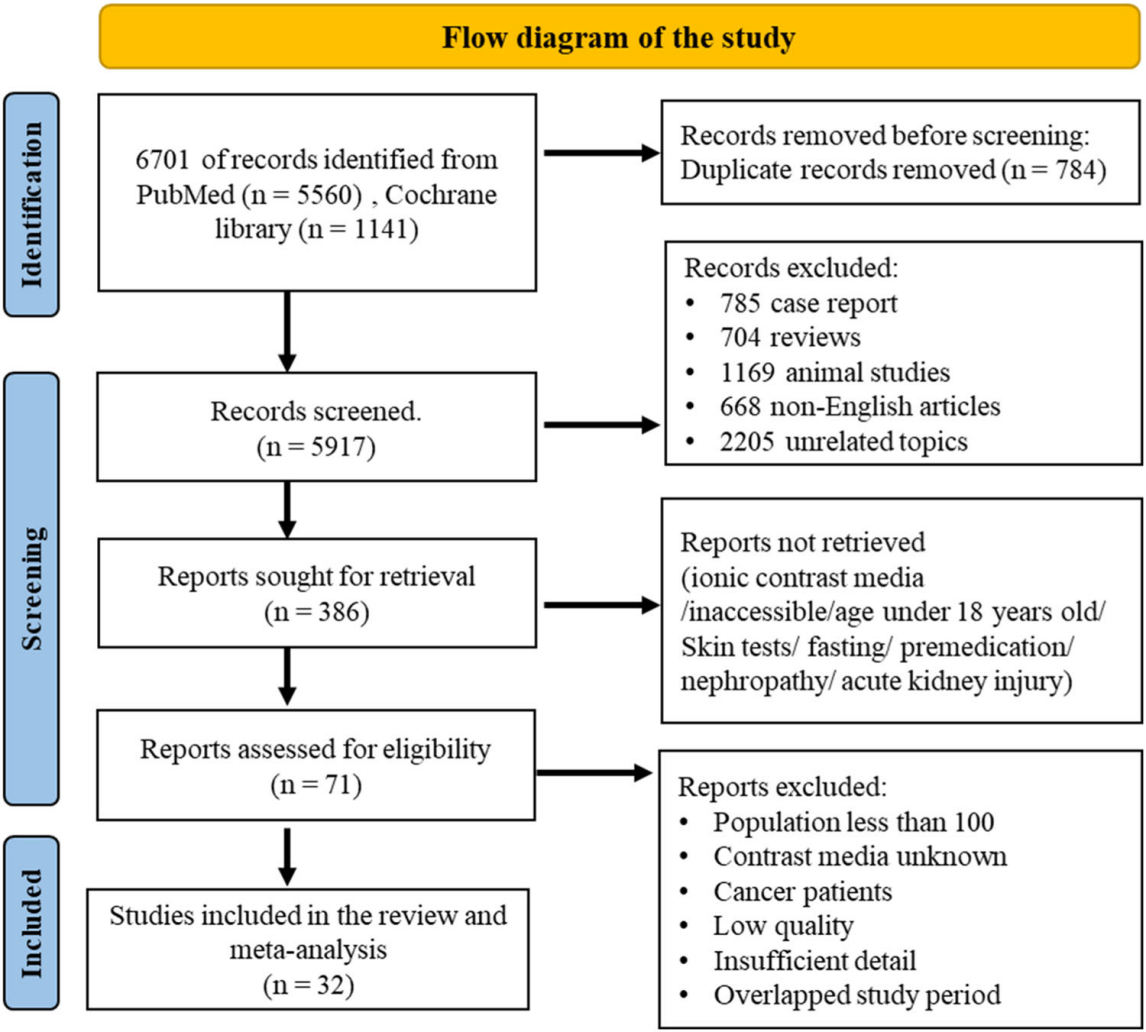
PRISMA flow diagram of study selection

After removing duplicates from 6701 initial database references (PubMed, *n* = 5560; Cochrane, *n* = 1141), 5917 abstracts were screened; 5531 articles were excluded due to non-compliance with the study selection criteria. 386 articles were considered for further assessment. After carefully evaluating and excluding studies not abiding by the inclusion criteria, 32 studies were included in the final review and meta-analysis (Fig. 1).

#### Characteristics of studies included

The characteristics of the included studies were carefully evaluated and summarized (Supplementary Table S1). The route of administration was IA for 6 studies, IV for 19 studies, and IA/IV for 7 studies. Single-center settings were utilized in 18 studies, while 14 employed multi-center settings. Four studies reported HSR only, while 28 reported HSR plus physiological reactions.

#### Quality of studies included

Among the 32 studies evaluated using the Newcastle Ottawa Scale, 12 studies had a good quality score (7), 15 studies had a fair quality score (6), and 5 studies were considered fair quality with a score of 5. Hence, the overall quality of the included studies was assessed as high (indicating a low bias), demonstrating good methodological rigor and reliable results (Supplementary Table S2).

### Pooled AAR rates associated with specific LOCM types

**Fig. 2:**
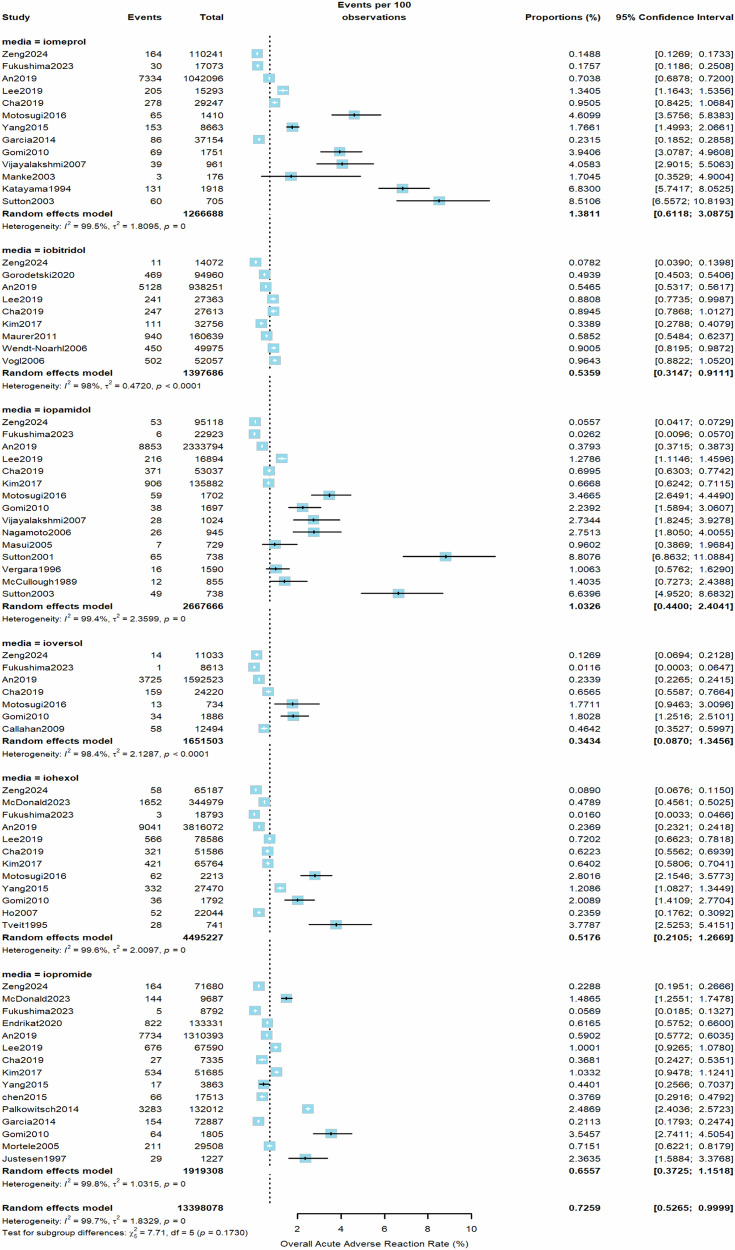
The pooled rate of overall AARs to the six LOCM from all included studies (random effects model)

**Fig. 3:**
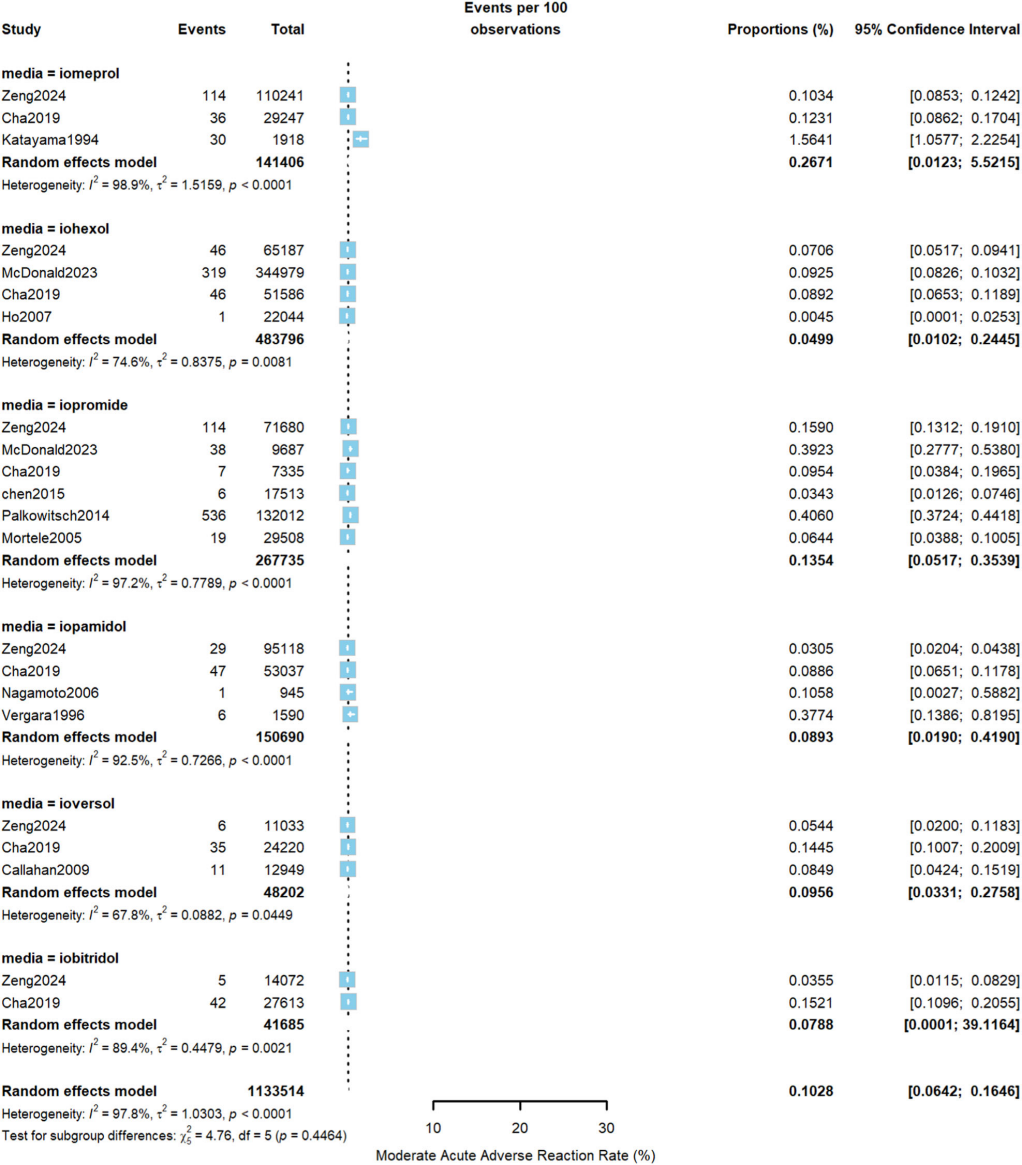
The pooled rate of moderate AARs according to the LOCM type (random effects model)

**Fig. 4:**
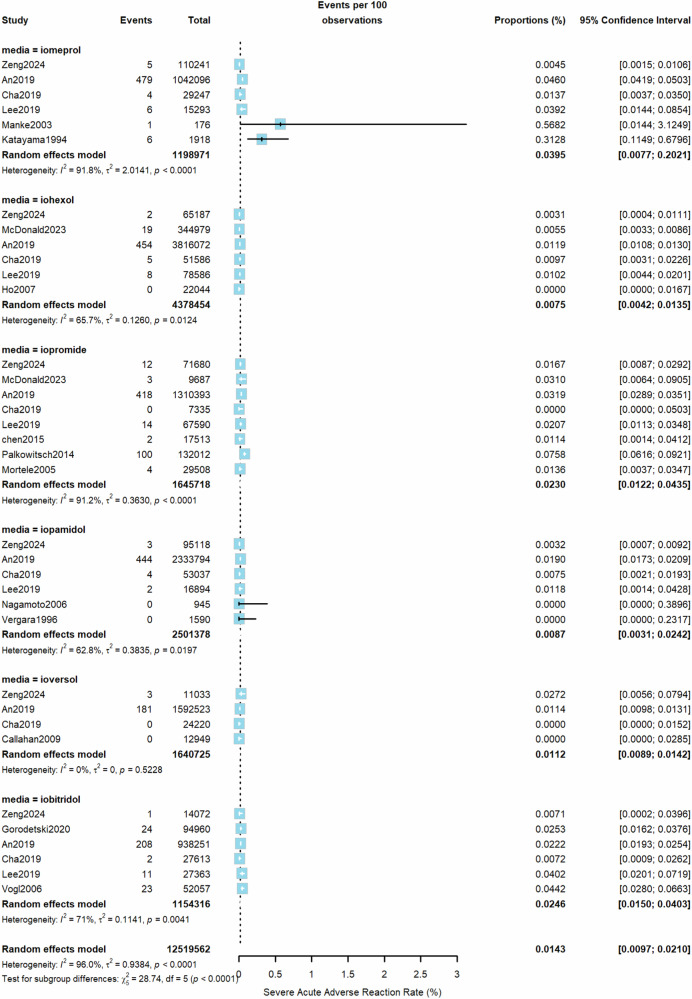
The pooled rate of severe AARs according to the LOCM type (random effects model)

From all included studies, the estimated pooled rate (random effects model) of overall AARs to the 6 LOCM was 0.73% (95% CI, 0.53–1.00%, *I*^2^ = 100%, τ^2^ = 1.7, *p* = 0) (Fig. 2). Iomeprol had the highest overall AAR rate at 1.38% (95% CI, 0.61–3.09%), while ioversol had the lowest at 0.34% (95% CI, 0.09–1.35%) compared to other LOCM (Table 1). For moderate AARs, the pooled rate (random effects model) from all studies was 0.10% (95% CI, 0.06–0.16%, *I*^2^ = 98%, τ^2^ = 1.03, *p* < 0.01, Fig. 3). Iomeprol had the highest moderate AAR rate at 0.27% (95% CI, 0.01–5.52%), while iohexol exhibited the lowest rate at 0.05% (95% CI, 0.01–0.24%, Table 1). The pooled rate (random effects model) of severe AARs to the 6 LOCM was 0.014% (95% CI, 0.0097–0.021%, *I*^2^ = 96%, τ^2^ = 0.94, *p* < 0.01, Fig. 4). Iomeprol showed the highest severe AAR rate at 0.040% (95% CI, 0.008–0.202%), while iohexol had the lowest severe AAR rate at 0.008% (95% CI, 0.004–0.014%, Table 1). The test for residual heterogeneity was significant for all moderators (*p* < 0.0001). Multivariable meta-regression analysis identified iomeprol as having the highest odds ratio for overall AARs, while iohexol and ioversol had the lowest (*p* < 0.0001, iomeprol as the baseline agent, Supplementary Table S3).

**Table 1:** Pooled AARs of the six LOCM

LOCM	Overall AAR [95% CI]	Moderate AAR [95% CI]	Severe AAR [95% CI]
Iomeprol	1.38% (0.61–3.09%)	0.27% (0.01–5.52%)	0.040% (0.008–0.202%)
Iopamidol	1.03% (0.44–2.40%)	0.09% (0.02–0.42%)	0.009% (0.003–0.024%)
Iobitridol	0.54% (0.31–0.91%)	0.08% (0.0001–39.12%)	0.025% (0.015–0.040%)
Iopromide	0.66% (0.37–1.15%)	0.14% (0.05–0.35%)	0.023% (0.012–0.043%)
Iohexol	0.52% (0.21–1.27%)	0.05% (0.01–0.24%)	0.008% (0.004–0.014%)
Ioversol	0.34% (0.09–1.35%)	0.10% (0.03–0.28%)	0.011% (0.009–0.014%)

*AAR* acute adverse reaction, *CI* confidence interval, *LOCM* low-osmolar contrast media

### Association of AARs with other factors

For overall AARs, apart from LOCM type, injection route, and study design were significantly associated with increased rates (*p* < 0.0001, *p* = 0.034, and *p* = 0.0001, respectively, Supplementary Table S3). No association was found between the rate of AARs and study location (center or country) (Table 2 and Supplementary Tables S3–S5). Retrospective studies reported lower overall AAR rates (0.41%) compared to prospective studies (2.45%) (Table 3). As regards differences between LOCM, iomeprol had the highest overall (0.72%) and severe AAR rates (0.031%) in retrospective studies while ioversol had the lowest rates (0.18%). In prospective studies, iomeprol displayed the highest AAR rate (4.52%). IA administration was linked to a higher overall and severe AAR rate (3.13% and 0.0439%, respectively) compared to IV administration (0.53% and 0.0076%; Table 2). Differences based on injection routes were significant across all severity categories (Supplementary Tables S3–S5 and Figs. S1–S3). The overall HSR rate was 0.39%, compared to 0.91% for HSR plus physiological reactions, with similar rates for moderate and severe reactions in both categories (0.113% vs 0.098% and 0.0125% vs 0.0157%, respectively, Table 2).

**Table 2:** Subgroup analysis based on injection route, study center, and hypersensitivity/physiological reactions

Subgroups (no. of studies)	Overall AAR [95% CI]	Moderate AAR [95% CI]	Severe AAR [95% CI]
Mode of administration			
IA (6 studies)	3.13% (1.34–7.14%)	0.034% (0.013–0.075%)	0.0439% (0.0000–99.99%)
IV (19 studies)	0.53% (0.35–0.82%)	0.089% (0.061–0.13%)	0.0076% (0.0045–0.0127%)
IA and IV comparison (7 studies)	0.82% (0.55–1.22%)	0.77% (0.0018–76.81%)	0.0269% (0.0170–0.0425%)
Study center			
Single-center (18 studies)	0.90% (0.57–1.43%)	0.089% (0.027–0.290%)	0.0125% (0.0067–0.0234%)
Multi-center (14 studies)	0.53% (0.35–0.80%)	0.108% (0.063–0.187%)	0.0153% (0.0093–0.0252%)
Hypersensitivity/physiological reactions			
HSR (4 studies)	0.39% (0.19–0.78%)	0.113% (0.087–0.145%)	0.0125% (0.0072–0.0216%)
HSR+ physio (28 studies)	0.91% (0.64–1.29%)	0.098% (0.050–0.193%)	0.0157% (0.0094–0.0262%)

*AAR* acute adverse reaction, *CI* confidence interval, *HSR* hypersensitivity reaction, *IA* intra-arterial, *IV* intravenous, *Physio* physiological reaction

**Table 3:** Subgroup analysis based on study design

LOCM	Retrospective		Prospective	
	Overall AAR [95% CI]	Severe AAR [95% CI]	Overall AAR [95% CI]	Severe AAR [95% CI]
Iomeprol	0.72% (0.25–2.01%)	0.031% (0.005–0.171%)	4.52% (2.75–7.34%)	NA
Iopamidol	0.27% (0.06–1.17%)	0.009% (0.0025–0.032%)	2.62% (1.48–4.58%)	NA
Iobitridol	0.54% (0.31–0.91%)	0.025% (0.015–0.040%)	NA	NA
Iopromide	0.52% (0.27–1.00%)	0.028% (0.013–0.057%)	1.21% (0.29–4.98%)	NA
Iohexol	0.30% (0.12–0.74%)	0.008% (0.0042–0.014%)	2.70% (1.50–4.81%)	NA
Ioversol	0.18% (0.04–0.87%)	0.011% (0.0082–0.016%)	1.79% (0.28–10.60%)	NA
All	0.41% (0.29–0.58%)	0.015% (0.010–0.022%)	2.45% (1.75–3.42%)	NA

*AAR* acute adverse reaction, *CI* confidence interval, *LOCM* low-osmolar contrast media, *NA* not available due to lack of data source

### Publication bias

The funnel plot was examined for asymmetry to assess the potential publication bias in the association between various LOCM types and the overall AAR rates. The analysis found no significant asymmetry, indicating the risk of publication bias to be low. This suggests that the included studies likely provide an unbiased estimate of the true association between different LOCM types and the rate of AARs (Supplementary Fig. S4). The sensitivity analysis illustrated that the *I*^2^ values were almost the same across studies and none of the omitted studies distorted the estimation and precision. The forest plot was ordered (low to high) by *I*^2^ (Supplementary Fig. S5).

## Discussion

Despite the overall low rate of AARs following ICM administration, they remain a significant concern for patient safety. With more than 120 million contrast-enhanced CT scans performed globally each year [10], even low AAR rates tend to affect many patients. Previous studies have reported varying AAR rates, as low as 0.15% in 298,491 patients [11] to higher rates ranging from 0.6% to 3.13% [12]. Over time, AARs have considerably reduced (0.2%–0.4%) due to the increased use of non-ionic agents [5]. In a recent study from South Korea, the AAR rate for non-ionic LOCM was reported to be 1.26% [13]. Our findings in this meta-analysis confirm these low overall AAR rates for LOCM but indicate up to 4-fold difference between agents, ranging from 0.34% for ioversol to 1.38% for iomeprol; and up to 5-fold difference for severe AARs ranging from 0.008% for iohexol to 0.040% for iomeprol. While a former meta-analysis showed no significant differences in AAR rates among contrast agents, several studies have demonstrated higher rates for iomeprol and iopromide consistent with our findings [4, 14, 15]. Significant statistical differences were also documented among ICM groups (ionic only, selective non-ionic use, and non-ionic only; *p* < 0.001) [16].

Looking at HSR only, the overall rate in our meta-analysis was 0.39%, with moderate and severe HSRs at 0.113% and 0.0125%, respectively, consistent with a recent meta-analysis (0.2–0.7%) [17]. Studies from Japan and Korea exhibited similar rates of severe HSRs at 0.04% and 0.01%, respectively [12, 18]. Some studies found a higher proportion of severe reactions and anaphylaxis risk for iomeprol [14, 19–22], or iopromide [23] while others did not demonstrate significant differences among non-ionic LOCM [13, 24]. Usually, the overall HSR rate is lower than the overall AAR (HSR plus physiological reactions) rate, whereas the severe rates are more similar. This is consistent with the fact that severe reactions are more frequent due to hypersensitivity. However, a LOCM comparison of HSR could not be performed due to the limited number of studies reporting HSR only. In addition, our findings suggested that the injection route impacted the AAR rate, with IV administration causing lower overall and severe AARs. Though non-apparent differences in patient demographics with IA vs IV procedures may have influenced the observed differences, this finding should be interpreted with caution, since evidence on the impact of injection route is heterogeneous, and no studies have directly compared administration routes.

This study demonstrates several key strengths that enhance its relevance. It includes an exceptionally large sample size, far exceeding those of previous meta-analyses (almost ten times), providing a more reliable outcome with higher statistical power for its findings, enabling more precise risk estimation. It offers a comprehensive examination of AAR severity, encompassing overall, moderate, and severe cases, ensuring a thorough exploration of the subject. The study also conducts extensive subgroup analyses, considering factors such as LOCM type, injection route, study design, and single- or multi-center settings. Subgroup analyses helped to identify sources of heterogeneity, while temporal trend analysis revealed research trajectories. Finally, a major strength of this systematic review and meta-analysis is that it combined data from studies across Western and Asian countries. No differences were observed across these countries, which enhances the global applicability of the results. These strengths make the study highly robust and impactful.

This study has certain limitations such as variations in study designs, patient populations, dosing regimens, and reporting methods which may have contributed to heterogeneity in the findings. Reliance on spontaneous reporting systems may have underestimated mild AARs. Patient risk factors for AARs (history of allergies, previous contrast reactions, asthma, renal insufficiency, and cardiac disease [18, 25–27]), the foremost being the history of a previous reaction to ICM [18], and the fact that switching agents upon re-exposure is considered the best preventive measure to reduce the risk of HSRs as advocated by the American College of Radiology (ACR) guidelines [27], might have impacted our analyses. This study also lacks subgroup analyses on prior ICM exposure, cross-reactivity reactions, and the potential impact of premedication. Additionally, a potentially decreased rate of AARs following the practice change of switching ICM upon re-exposure is uncertain since the culprit is often unknown [28]. Different ICMs used in various settings based on institutional policies may have biased the results. Patients’ stress could also have contributed to AAR rate differences, though linking increased stress to higher AAR rates is challenging. Finally, our study could not compare the different LOCM for HSR vs HSR plus physiological AAR rates since most included studies reported HSR plus physiological reactions (28/32 studies), the latter being dose-dependent, hence higher doses of LOCM (concentration and/or volume) may have influenced the overall AAR rates.

In conclusion, among the LOCM studied, iohexol and ioversol had the lowest severe and overall AAR rates, while iomeprol had the highest. Based on a significantly large dataset and rigorous analysis of AAR severity, along with extensive sub-analyses, this meta-analysis provides additional evidence for significant differences in safety profiles within the class of non-ionic LOCM, particularly for moderate and severe AARs. Considering the huge number of contrast-enhanced CT examinations performed worldwide yearly, these findings can guide clinicians in selecting contrast agents with the aim to further reduce the risk of AARs and improving patient safety in diagnostic imaging procedures across various clinical settings. Regardless of the advances in detection, prevention, and treatment, AARs continue to remain a clinical challenge, warranting ongoing vigilance and research.

## Supplementary information

ELECTRONIC SUPPLEMENTARY MATERIAL

## Abbreviations

AAR: Acute adverse reactions
CI: Confidence interval
CT: Computed tomography
HOCM: High-osmolar contrast media
HSR: Hypersensitivity reactions
IA: Intra-arterial
ICM: Iodinated contrast media
IV: Intravenous
LOCM: Low-osmolar contrast media
NA: Not available due to lack of data source
PRISMA: Preferred reporting items for systematic reviews and meta-analyses
