# Supplementary material for: Estimating the rate of acute adverse reactions to non-ionic low-osmolar contrast media: a systematic review and meta-analysis
Source: Eur Radiol. 2025 Apr 11;35(10):6240–9. doi: 10.1007/s00330-025-11526-z (PMC12417258; doi:10.1007/s00330-025-11526-z)
Supplement: Supplementary file 1 — ELECTRONIC SUPPLEMENTARY MATERIAL [file 330_2025_11526_MOESM1_ESM.pdf]

Estimating the Rate of Acute Adverse Reactions to Non-Ionic Low-Osmolar Contrast Media: A Systematic Review and Meta-Analysis

ELECTRONIC SUPPLEMENTARY MATERIAL

Eur Radiol (2025) Wei Y, Jian X, Hibberd M, Sampedro A, Rautenbach J.

Supplementary Table S1 Characteristics of the included studies

Source	Country	Study period	Type of examinations	Study design	Number of patients	Injection route	Age (in years)	Sex (% of males)	Single-/multi-center	Physiological reactions (yes/no)	AAR/HSR
Fukushima 2023 [20]	Japan	2016.04-2021.09	CT	Retrospective	76,194	IV	68 (58, 75)	58%	Single-center	No	HSR
An 2019 [19]	Korea	2014.01-2016.12	CT	Retrospective	11,712,796	IA or IV	53.9 (14.0)	46.90 %	Multi-center	No	HSR_physio
Cha 2019 [18]	Korea	2017.03-2017.10	CT	Observational study	196,081	IV	59.1 (16.06)	53.60 %	Multi-center	No	HSR
Lee 2019 [29]	Korea	2012-2014	CT	Observational- longitudinal study	205,726	IA or IV		NR	Single-center	No	HSR
Kim 2017 [30]	Korea	2006.01-2010.12	CT	Retrospective	142,099	IV	51.60 (18.50)	NR	Single-center	No	HSR_physio
Motosugi 2016 [31]	Japan	2012.02-2013.03	CT	RCT	5,959	IV	NR	NR	Single-center	Yes	HSR_physio
Yang 2015 [32]	Korea	2011.06-2012.05	CT	Retrospective	40,052	IA or IV	52.6 (14.0) / 56.8 (16.3) (occurred / total)	54.80 %	Single-center	No	HSR
Garcia 2014 [21]	Spain	1997-2006 (iopromide) 2009.01-2013.04 (iomeprol)	CT & Urography	Retrospective	72,887 iopromide 37,154 iomeprol	IV	50.96 (17.9) (iopromide) 55.46 (17.5) (iomeprol)	NR	Single-center	No	HSR_physio
Gomi 2010 [14]	Japan	2004.06-2007.05	CT	Prospective	8,931	IV	NR	NR	Single-center	No	HSR_physio
Vijayalakshmi 2007 [33]	UK	2001.06-2002.05	Coronary angiography, Left	Prospective	1,985	IA	NR	NR	Single-center	Yes	HSR_physio

Source	Country	Study period	Type of examinations	Study design	Number of patients	Injection route	Age (in years)	Sex (% of males)	Single-/multi-center	Physiological reactions (yes/no)	AAR/HSR
			ventriculography & aortography								
Ho 2007 [34]	Canada	2002-2004	CT	Retrospective	37,187	IV	NR	53%	Single-center	No	HSR_physio
Nagamoto 2006 [35]	Japan	2002-2003	CT	Prospective	945	IV	64.1	60.20 %	Single-center	Yes	HSR_physio
Wendt-Noarhl 2006 [36]	Germany	NR	Urography	Retrospective	49,975	IV	56.6	56.70 %	Multi-center	No	HSR_physio
Masui 2005 [37]	Japan	2002.02-2002.03	CT	Prospective	729	IV	59.8 (14.1) 61.7 (13.5)	60.10 %	Single-center	Yes	HSR_physio
Sutton 2001 [38]	UK	1997.02-1998.01	DSA	Prospective	2,018	IA	60.3(9.9)	66%	Single-center	Yes	HSR_physio
Sutton 2003 [39]	UK	1998.04-1999.04	DSA	Prospective	2,108	IA	60.4(10.3)	65.50 %	Single-center	Yes	HSR_physio
Manke 2003 [40]	Europe	2000.03-2001.03	DSA	Prospective	352	IA	63.6 (11.2) 65.2 (11.6)	17.70 %	Multi-center	No	HSR_physio
Justesen 1997 [41]	Europe	1994.4-1994.11	DSA	Prospective	2,372	IA	65.6 (11.5) (iodixanol) 65.0 (11.3) (iopromide)	72.20 %	Multi-center	No	HSR_physio
Vergara 1996 [42]	Chile	NR	CT	Prospective	1,590	IV	33.5 (4.3-66.1)	43.40 %	Single-center	No	HSR_physio
Tveit 1995 [43]	Europe	NR	Urography	Prospective	1,481	IV	51.1 (17-90)	60.6%	Multi-center	No	HSR_physio
Katayama 1994 [15]	Japan	1990-1992	DSA /Urography/ CT	Retrospective	1,918	IA or IV	NR	61.40 %	Multi-center	No	HSR_physio
McCullough 1989 [44]	UK	NR	Urography	Prospective	855	IV	NR	NR	Single-center	No	HSR_physio

Source	Country	Study period	Type of examinations	Study design	Number of patients	Injection route	Age (in years)	Sex (% of males)	Single-/multi-center	Physiological reactions (yes/no)	AAR/HSR
Zeng 2024 [45]	China	2017.01.01-2021.12.31	CT	Retrospective	473,482	IV	55.22 (14.85)	253,499 (53.54%)	Multi-center	No	HSR_physio
McDonald 2023 [46]	USA	2009.06.01-2017.05.09	CT	Retrospective	359,997	IV	61 (49-72)	53%	Single-center	Yes	HSR_physio
Endrikat 2020 [47]	Europe+Asia	1999-2011	CT+ intervention	Retrospective	133,331	IA or IV	NR	NR	Multi-center	No	HSR_physio
Gorodetski 2020 [48]	Germany	2009-2013	CT + intervention	Retrospective	94,960	IA or IV	58.8	49,819 (53.1%)	Multi-center	No	HSR_physio
Chen 2015 [49]	China	2010.08-2011.09	CAG or PCI	Prospective	17,513	IA	60.3 (10.5)	66%	Multi-center	No	HSR_physio
Palkowitsch2014 [50]	Global	NR	CT + intervention	Retrospective	132,012	IA or IV	57	70,911 (53.7%)	Multi-center	No	HSR_physio
Maurer 2011 [51]	Germany	2002.01-2008.12	CT	Retrospective	160,639	IV	58.6	55.10%	Multi-center	No	HSR_physio
Callahan 2009 [52]	USA	1999.01-2005.12	CT or Excretory Urographic	Retrospective	12,494	IV	9.5 (5.9)	52.83%	Single-center	No	HSR_physio
Vogl 2006 [53]	Germany	2000.07-2004.03	CT	Retrospective	52,057	IV	58.96	49.55%	Multi-center	No	HSR_physio
Mortele 2005 [54]	USA	2001.06-2003.05	CT	Prospective	29,508	IV	54.6	43%	Single-center	No	HSR_physio

AAR acute adverse reaction, *CAG* coronary angiography, *CT* s, *DSA* digital subtraction angiography, *IA* intra-arterial, *IV* intravenous, *HSR* hypersensitivity reaction, *NR* not reported, *PCI* percutaneous coronary intervention, *Physio* physiological reaction, *RCT* randomized controlled trial. The age (in years) is presented as mean (SD) or median (interquartile range) as reported in the included articles.

Supplementary Table S2 Quality assessment of the included studies using the Newcastle Ottawa Scale

Author & year	Representativeness of the exposed cohort	Selection of the non-exposed cohort	Ascertainment of exposure	Demonstration that outcome of interest was not present at the start of the study	Comparability of cohorts based on the design or analysis	Assessment of outcome	Was follow-up long enough for outcomes to occur	Adequacy of follow-up of cohorts	Total score
Fukushima 2023 [20]	*	—	*	*	**	*	—	*	7
An 2019 [19]	*	—	*	*	**	*	—	*	7
Cha 2019 [18]	*	—	*	*	**	*	—	*	7
Lee 2019 [29]	*	—	*	*	*	*	—	*	6
Kim 2017 [30]	*	—	*	*	**	*	—	*	7
Motosugi 2016 [31]	*	—	*	*	**	*	—	*	7
Yang 2015 [32]	*	—	*	*	**	*	—	*	7
García 2014 [21]	*	—	*	*	*	*	—	*	6
Gomi 2010 [14]	*	—	*	*	*	*	—	*	6
Vijayalakshmi 2007 [33]	*	—	*	*	*	*	—	*	6
Ho 2007 [34]	*	—	*	*	*	*	—	*	6
Nagamoto 2006 [35]	*	—	*	*	*	*	—	*	6
Wendt-Nordahl 2006 [36]	*	—	—	*	*	*	—	*	5
Masui 2005 [37]	*	—	*	*	*	*	—	*	6
Sutton 2001 [38]	*	—	*	*	**	*	—	*	7
Sutton2003 [39]	*	—	*	*	* *	*	—	*	7
Manke 2003 [40]	*	—	*	*	*	*	—	*	6
Justesen 1997 [41]	*	—	*	*	*	*	—	*	6

Author & year	Representativeness of the exposed cohort	Selection of the non-exposed cohort	Ascertainment of exposure	Demonstration that outcome of interest was not present at the start of the study	Comparability of cohorts based on the design or analysis	Assessment of outcome	Was follow-up long enough for outcomes to occur	Adequacy of follow-up of cohorts	Total score
Vergara 1996 [42]	*	—	*	*	—	*	—	*	5
Tveit 1995 [43]	*	—	*	*	*	*	—	*	6
Katayama 1994 [15]	*	—	*	*	*	*	—	*	6
McCullough 1989 [44]	*	—	*	*	—	*	—	*	5
Zeng 2024 [45]	*	—	*	*	**	*	—	*	7
McDonald 2023 [46]	*	—	*	*	**	*	—	*	7
Endrikat 2020 [47]	*	—	*	*	**	*	—	*	7
Gorodetski 2020 [48]	*	—	*	*	**	*	—	*	7
Chen 2015 [49]	*	—	*	*	*	*	—	*	6
Palkowitsch 2014 [50]	*	—	—	*	**	*	—	*	6
Maurer 2011[51]	*	—	*	*	*	*	—	*	6
Callahan 2009 [52]	*	—	*	*	—	*	—	*	5
Vogl 2006 [53]	*	—	*	*	*	*	—	*	6
Mortelé 2005 [54]	*	—	*	*	—	*	—	*	5

ICM iodinated contrast media, *KAERS* Korea adverse event reporting system

Supplementary Table S3 Meta-regression analysis of overall AAR

Variables		Univariable meta-regression analysis			Multivariable meta-regression analysis		
		OR [95% CI]	*p*	Test for residual heterogeneity	OR [95% CI]	*p*	Test for residual heterogeneity
LOCM	Iomeprol	1	--------	$p < 0.0001$	1	--------	$p < 0.0001$
	Iohexol	0.51 [0.43, 0.62]	< 0.0001		0.53 [0.42, 0.66]	< 0.0001	
	Iopamidol	0.66 [0.57, 0.77]	< 0.0001		0.68 [0.55, 0.84]	0.0004	
	Iopromide	0.69 [0.54, 0.88]	0.0028		0.70 [0.51, 0.96]	0.0274	
	Ioversol	0.48 [0.38, 0.60]	< 0.0001		0.49 [0.40, 0.60]	< 0.0001	
	Iobitridol	0.38 [0.25, 0.58]	< 0.0001		0.71 [0.57, 0.89]	0.0030	
Injection route	IA	1	--------	$p < 0.0001$			
	IV	0.27 [0.10, 0.73]	0.0103		--------	--------	--------
	IA or IV	0.42 [0.13, 1.29]	0.1284				
Study design	Prospective	1	--------		1	--------	
	Retrospective	0.28 [0.14, 0.53]	0.0001	$p < 0.0001$	0.22 [0.14, 0.35]	< 0.0001	--------
Center	Single	1	--------				
	Multi	0.858 [0.40, 1.86]	0.698	$p < 0.0001$	--------	--------	--------
Country	Asian	1	--------				
	Western	1.28 [0.55, 2.97]	0.568	$p < 0.0001$	--------	--------	--------
	Global	1.38 [0.42, 4.49]	0.593				

CI confidence interval, *IA* intra-arterial, *IV* intravenous, *OR* odds ratio

Supplementary Table S4 Meta-regression analysis of moderate AAR

Variables		Univariable meta-regression analysis			Multivariable meta-regression analysis		
		**OR [95%CI]**	***p***	**Test for residual heterogeneity**	**OR [95%CI]**	***p***	**Test for residual heterogeneity**
LOCM	Iomeprol	1		$p < 0.001$	1		$P < 0.001$
	Iohexol	0.35 [0.13, 0.93]	0.0354		0.63 [0.48, 0.84]	0.0017	
	Iopamidol	0.41 [0.24, 0.73]	0.0022		0.64 [0.30, 1.36]	0.2484	
	Iopromide	0.80 [0.46, 1.39]	0.4267		0.73 [0.27, 1.51]	0.3929	
	Ioversol	0.42 [0.14, 1.27]	0.1246		1.03[0.74, 1.82]	0.9256	
	Iobitridol	0.58 [0.27, 1.25]	0.1657		1.04 [0.54, 2.96]	0.9370	
Injection route	IA	1		$p < 0.001$			
	IV	3.07 [1.38, 6.85]	0.0062		2.61[0.98, 6.95]	0.0542	
	IA or IV	22.94 [6.67, 78.92]	< 0.0001		32.5[10.58, 99.99]	< 0.0001	
Study design	Prospective	1		$p < 0.001$			
	Retrospective	1.47 [0.36, 6.04]	0.5926				
center	single	1		$p < 0.001$			
	multi	2.242 [0.57, 8.78]	0.2462				
Country	Asian	1		$p < 0.001$			
	Western	0.54 [0.12, 2.39]	0.4185				
	Global	2.68 [0.82, 8.80]	0.1043				

CI confidence interval, *IA* intra-arterial, *IV* intravenous, *OR* odds ratio

Supplementary Table S5 Meta-regression analysis of severe AAR

Variables		Univariable meta-regression analysis			Multivariable meta-regression analysis		
		**OR [95%CI]**	***p***	**Test for residual heterogeneity**	**OR [95%CI]**	***p***	**Test for residual heterogeneity**
LOCM	Iomeprol	1		$p < 0.0001$	1		$P < 0.0001$
	Iohexol	0.24 [0.14, 0.41]	< 0.0001		0. 25 [0.12, 0.51]	0.0001	
	Iopamidol	0.39 [0.29, 0.53]	< 0.0001		0.40 [0.28, 0.56]	< 0.0001	
	Iopromide	0.62 [0.32, 1.20]	0.1558		0.64 [0.27, 1.52]	0.3128	
	Ioversol	0.22 [0.06, 0.77]	0.0180		0.24[0.05, 1.08]	0.0622	
	Iobitridol	0.53 [0.24, 1.18]	0.1184		0.57 [0.28, 1.15]	0.1163	
Injection route	IA	1		$p < 0.0001$			
	IV	0.18 [0.01, 2.81]	0.2203				
	IA or IV	0.67 [0.04, 11.29]	0.7799				
Study design	Prospective	1		$p < 0.0001$			
	Retrospective	0.62 [0.14, 2.74]	0.5294				
Center	Single	1		$p < 0.0001$			
	Multi	2.91 [1.06, 7.95]	0.0378		2.37[1.07, 5.22]	0.0324	
Country	Asian	1		$p < 0.0001$			
	Western	0.85[0.24, 3.00]	0.8028		1.24[0.48,3.21]	0.6576	
	Global	3.10 [1.24, 7.73]	0.0152		2.45[0.82, 7.26]	0.1067	

CI confidence interval, *IA* intra-arterial, *IV* intravenous, *OR* odds ratio

Study	Events	Total	Proportions (%)	95% Confidence Interval
**Injection.route = IV**				
Zeng2024	164	110241	0.1488	[0.1269; 0.1733]
Fukushima2023	30	17073	0.1757	[0.1186; 0.2508]
Cha2019	278	29247	0.9505	[0.8425; 1.0684]
Motosugi2016	65	1410	4.6099	[3.5756; 5.8383]
Garcia2014	86	37154	0.2315	[0.1852; 0.2858]
Gomi2010	69	1751	3.9406	[3.0787; 4.9608]
Zeng2024	11	14072	0.0782	[0.0390; 0.1398]
Cha2019	247	27613	0.8945	[0.7868; 1.0127]
Kim2017	111	32756	0.3389	[0.2788; 0.4079]
Maurer2011	940	160639	0.5852	[0.5484; 0.6237]
Wendt-Noarhl2006	450	49975	0.9005	[0.8195; 0.9872]
Vogl2006	502	52057	0.9643	[0.8822; 1.0520]
Zeng2024	53	95118	0.0557	[0.0417; 0.0729]
Fukushima2023	6	22923	0.0262	[0.0096; 0.0570]
Cha2019	371	53037	0.6995	[0.6303; 0.7742]
Kim2017	906	135882	0.6668	[0.6242; 0.7115]
Motosugi2016	59	1702	3.4665	[2.6491; 4.4490]
Gomi2010	38	1697	2.2392	[1.5894; 3.0607]
Nagamoto2006	26	945	2.7513	[1.8050; 4.0055]
Masui2005	7	729	0.9602	[0.3869; 1.9684]
Vergara1996	16	1590	1.0063	[0.5762; 1.6290]
McCullough1989	12	855	1.4035	[0.7273; 2.4388]
Zeng2024	14	11033	0.1269	[0.0694; 0.2128]
Fukushima2023	1	8613	0.0116	[0.0003; 0.0647]
Cha2019	159	24220	0.6565	[0.5587; 0.7664]
Motosugi2016	13	734	1.7711	[0.9463; 3.0096]
Gomi2010	34	1886	1.8028	[1.2516; 2.5101]
Callahan2009	58	12494	0.4642	[0.3527; 0.5997]
Zeng2024	58	65187	0.0890	[0.0676; 0.1150]
McDonald2023	1652	344979	0.4789	[0.4561; 0.5025]
Fukushima2023	3	18793	0.0160	[0.0033; 0.0466]
Cha2019	321	51586	0.6223	[0.5562; 0.6939]
Kim2017	421	65764	0.6402	[0.5806; 0.7041]
Motosugi2016	62	2213	2.8016	[2.1546; 3.5773]
Gomi2010	36	1792	2.0089	[1.4109; 2.7704]
Ho2007	52	22044	0.2359	[0.1762; 0.3092]
Tveit1995	28	741	3.7787	[2.5253; 5.4151]
Zeng2024	164	71680	0.2288	[0.1951; 0.2666]
McDonald2023	144	9687	1.4865	[1.2551; 1.7478]
Fukushima2023	5	8792	0.0569	[0.0185; 0.1327]
Cha2019	27	7335	0.3681	[0.2427; 0.5351]
Kim2017	534	51685	1.0332	[0.9478; 1.1241]
Garcia2014	154	72887	0.2113	[0.1793; 0.2474]
Gomi2010	64	1805	3.5457	[2.7411; 4.5054]
Mortele2005	211	29508	0.7151	[0.6221; 0.8179]
**Random effects model**		**1733924**	**0.5334**	**[0.3476; 0.8176]**
Heterogeneity: I^2 = 98.8%, τ^2 = 2.0047, p = 0				
**Injection.route = IA_IV**				
An2019	7334	1042096	0.7038	[0.6878; 0.7200]
Lee2019	205	15293	1.3405	[1.1643; 1.5356]
Yang2015	153	8663	1.7661	[1.4993; 2.0661]
Katayama1994	131	1918	6.8300	[5.7417; 8.0525]
Gorodetski2020	469	94960	0.4939	[0.4503; 0.5406]
An2019	5128	938251	0.5465	[0.5317; 0.5617]
Lee2019	241	27363	0.8808	[0.7735; 0.9987]
An2019	8853	2333794	0.3793	[0.3715; 0.3873]
Lee2019	216	16894	1.2786	[1.1146; 1.4596]
An2019	3725	1592523	0.2339	[0.2265; 0.2415]
An2019	9041	3816072	0.2369	[0.2321; 0.2418]
Lee2019	566	78586	0.7202	[0.6623; 0.7818]
Yang2015	332	27470	1.2086	[1.0827; 1.3449]
Endrikat2020	822	133331	0.6165	[0.5752; 0.6600]
An2019	7734	1310393	0.5902	[0.5772; 0.6035]
Lee2019	676	67590	1.0001	[0.9265; 1.0780]
Yang2015	17	3863	0.4401	[0.2566; 0.7037]
Palkowitsch2014	3283	132012	2.4869	[2.4036; 2.5723]
**Random effects model**		**11641072**	**0.8169**	**[0.5454; 1.2218]**
Heterogeneity: I^2 = 99.9%, τ^2 = 0.6652, p = 0				
**Injection.route = IA**				
Vijayalakshmi2007	39	961	4.0583	[2.9015; 5.5063]
Manke2003	3	176	1.7045	[0.3529; 4.9004]
Vijayalakshmi2007	28	1024	2.7344	[1.8245; 3.9278]
Sutton2001	65	738	8.8076	[6.8632; 11.0884]
chen2015	66	17513	0.3769	[0.2916; 0.4792]
Justesen1997	29	1227	2.3635	[1.5884; 3.3768]
Sutton2003	60	705	8.5106	[6.5572; 10.8193]
Sutton2003	49	738	6.6396	[4.9520; 8.6832]
**Random effects model**		**23082**	**3.1254**	**[1.3365; 7.1354]**
Heterogeneity: I^2 = 98.5%, τ^2 = 1.0231, p < 0.0001				
**Random effects model**		**13398078**	**0.7259**	**[0.5265; 0.9999]**
Heterogeneity: I^2 = 99.7%, τ^2 = 1.8329, p = 0				
Test for subgroup differences: χ^2_2 = 17.88, df = 2 (p = 0.0001)				

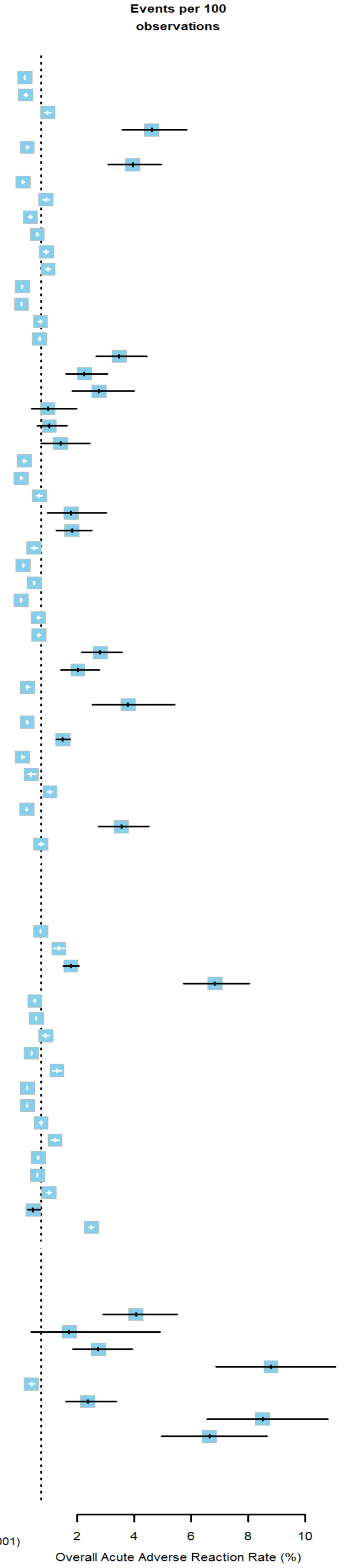

Supplementary Fig. S1 The pooled overall AARs of the injection route

Eur Radiol (2025) Wei Y, Jian X, Hibberd M, Sampedro A, Rautenbach J.

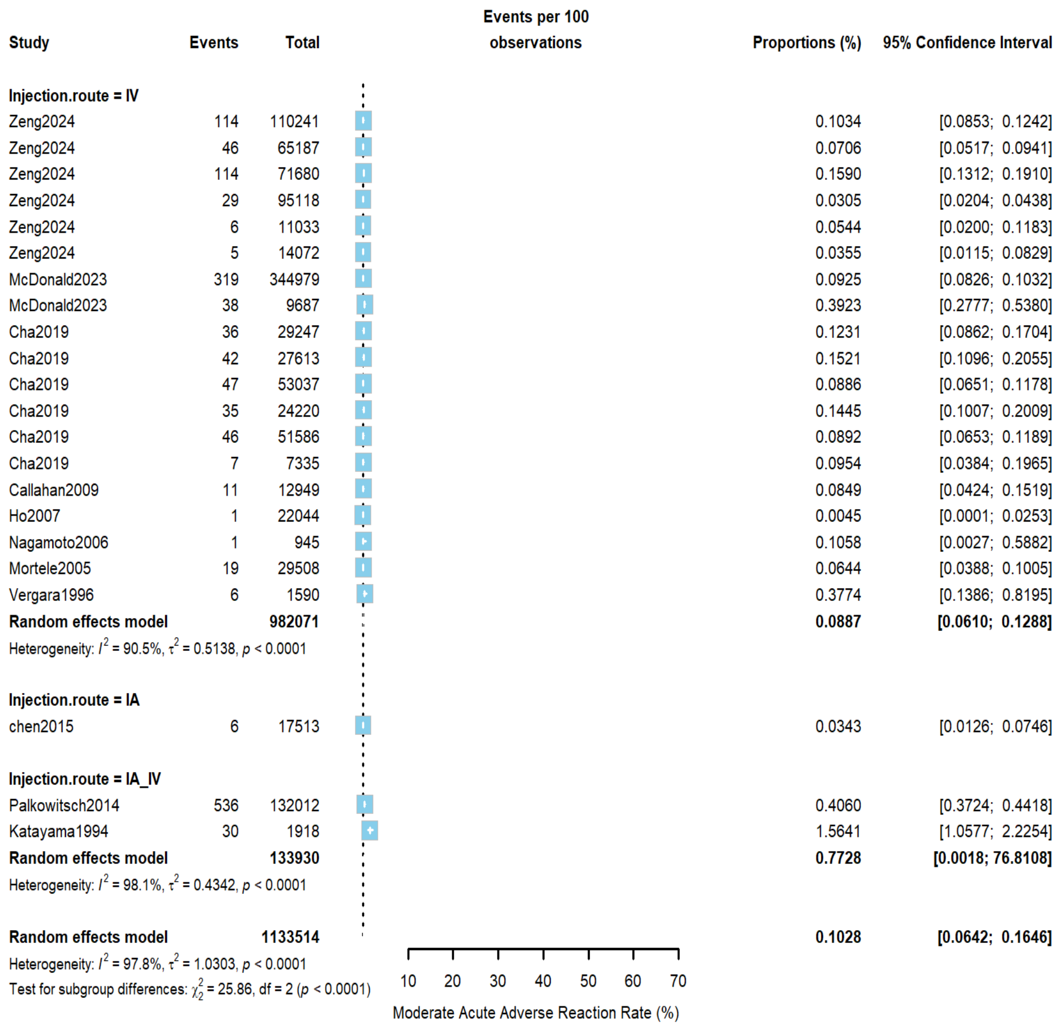

Supplementary Fig. S2 The pooled moderate AARs of the injection route

Study	Events	Total	Proportions (%)	95% Confidence Interval
**Injection.route = IV**				
Zeng2024	5	110241	0.0045	[0.0015; 0.0106]
Zeng2024	2	65187	0.0031	[0.0004; 0.0111]
Zeng2024	12	71680	0.0167	[0.0087; 0.0292]
Zeng2024	3	95118	0.0032	[0.0007; 0.0092]
Zeng2024	3	11033	0.0272	[0.0056; 0.0794]
Zeng2024	1	14072	0.0071	[0.0002; 0.0396]
McDonald2023	19	344979	0.0055	[0.0033; 0.0086]
McDonald2023	3	9687	0.0310	[0.0064; 0.0905]
Cha2019	4	29247	0.0137	[0.0037; 0.0350]
Cha2019	2	27613	0.0072	[0.0009; 0.0262]
Cha2019	4	53037	0.0075	[0.0021; 0.0193]
Cha2019	0	24220	0.0000	[0.0000; 0.0152]
Cha2019	5	51586	0.0097	[0.0031; 0.0226]
Cha2019	0	7335	0.0000	[0.0000; 0.0503]
Callahan2009	0	12949	0.0000	[0.0000; 0.0285]
Ho2007	0	22044	0.0000	[0.0000; 0.0167]
Nagamoto2006	0	945	0.0000	[0.0000; 0.3896]
Vogl2006	23	52057	0.0442	[0.0280; 0.0663]
Mortele2005	4	29508	0.0136	[0.0037; 0.0347]
Vergara1996	0	1590	0.0000	[0.0000; 0.2317]
**Random effects model**		**1034128**	**0.0076**	**[0.0045; 0.0127]**
Heterogeneity: $I^2 = 73.4\%$, $\tau^2 = 0.6016$, $p < 0.0001$				
**Injection.route = IA_IV**				
Gorodetski2020	24	94960	0.0253	[0.0162; 0.0376]
An2019	479	1042096	0.0460	[0.0419; 0.0503]
An2019	208	938251	0.0222	[0.0193; 0.0254]
An2019	444	2333794	0.0190	[0.0173; 0.0209]
An2019	181	1592523	0.0114	[0.0098; 0.0131]
An2019	454	3816072	0.0119	[0.0108; 0.0130]
An2019	418	1310393	0.0319	[0.0289; 0.0351]
Lee2019	6	15293	0.0392	[0.0144; 0.0854]
Lee2019	11	27363	0.0402	[0.0201; 0.0719]
Lee2019	2	16894	0.0118	[0.0014; 0.0428]
Lee2019	8	78586	0.0102	[0.0044; 0.0201]
Lee2019	14	67590	0.0207	[0.0113; 0.0348]
Palkowitsch2014	100	132012	0.0758	[0.0616; 0.0921]
Katayama1994	6	1918	0.3128	[0.1149; 0.6796]
**Random effects model**		**11467745**	**0.0269**	**[0.0170; 0.0425]**
Heterogeneity: $I^2 = 98.3\%$, $\tau^2 = 0.5525$, $p < 0.0001$				
**Injection.route = IA**				
chen2015	2	17513	0.0114	[0.0014; 0.0412]
Manke2003	1	176	0.5682	[0.0144; 3.1249]
**Random effects model**		**17689**	**0.0439**	**[0.0000; 99.9963]**
Heterogeneity: $I^2 = 90.2\%$, $\tau^2 = 2.5122$, $p = 0.0014$				
**Random effects model**		**12519562**	**0.0143**	**[0.0097; 0.0210]**
Heterogeneity: $I^2 = 96.0\%$, $\tau^2 = 0.9384$, $p < 0.0001$				
Test for subgroup differences: $\chi^2_2 = 15.84$, df = 2 ($p = 0.0004$)				

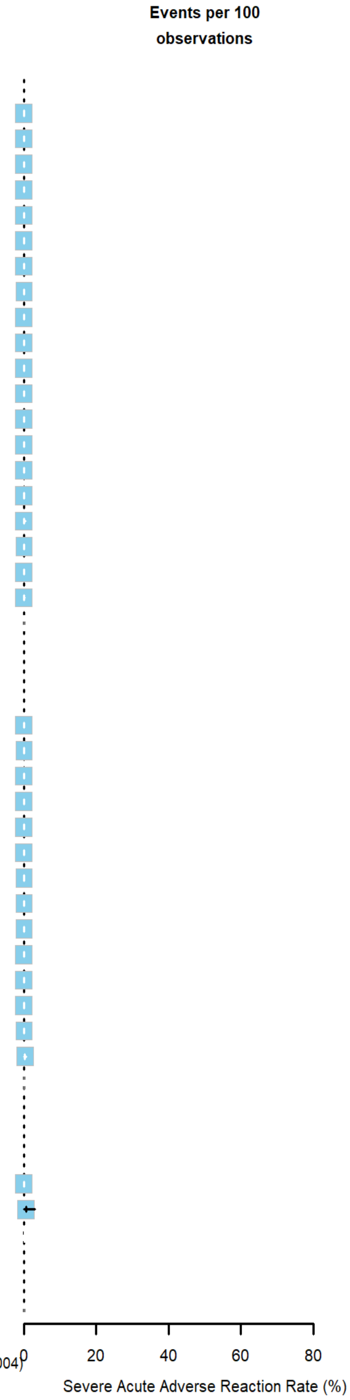

Supplementary Fig. S3 The pooled severe AARs of the injection route

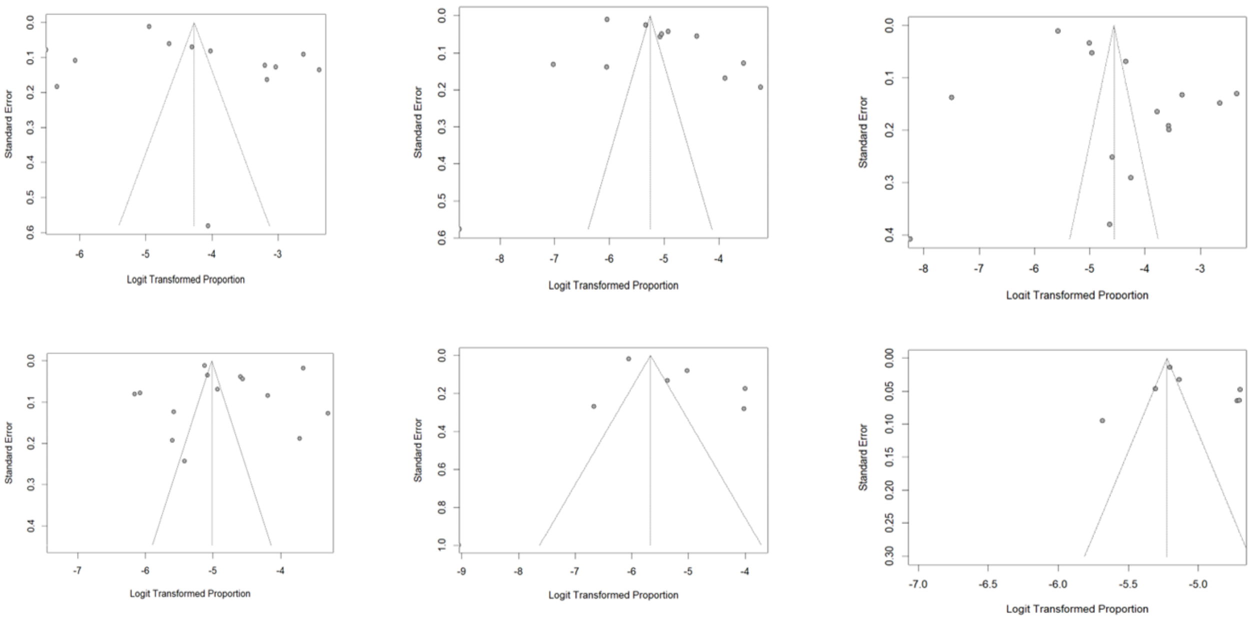

Supplementary Fig. S4 Funnel plots of overall AARs of 6 LOCM; the plots are presented for iomeprol, iohexol, iopamidol, iopromide, ioversol, and iobitridol respectively

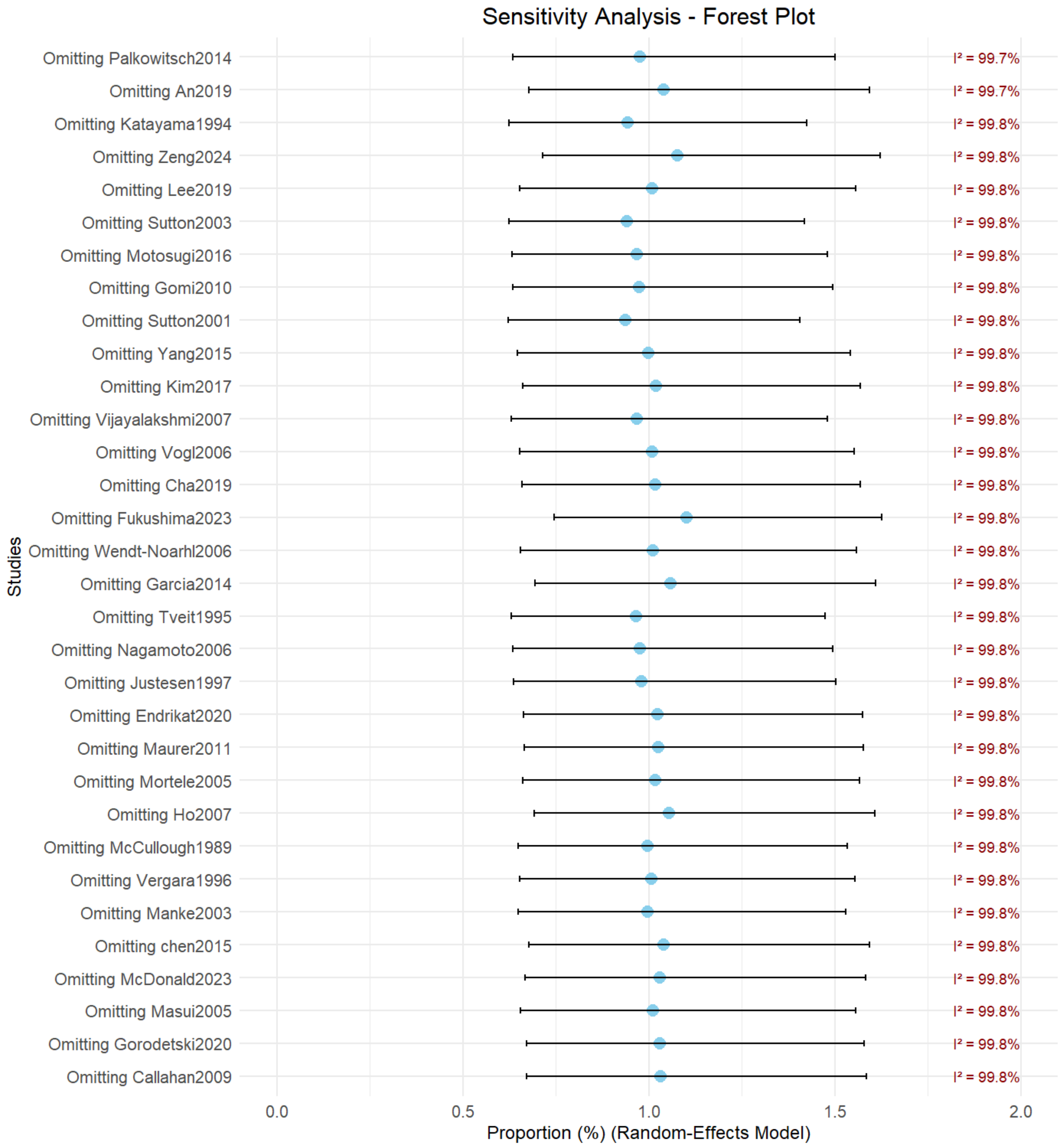

Supplementary Fig. S5 Sensitivity analysis of the included studies

29 Lee SY, Kang DY, Kim JY et al (2019) Incidence and Risk Factors of Immediate Hypersensitivity Reactions Associated With Low-Osmolar Iodinated Contrast Media: A Longitudinal Study Based on a Real-Time Monitoring System. J Investig Allergol Clin Immunol 29:444-450
30 Kim SR, Lee JH, Park KH, Park HJ, Park JW (2017) Varied incidence of immediate adverse reactions to low-osmolar non-ionic iodide radiocontrast media used in computed tomography. Clin Exp Allergy 47:106-112
31 Motosugi U, Ichikawa T, Sano K, &, Onishi H (2016) Acute adverse reactions to nonionic iodinated contrast media for CT: prospective randomized evaluation of the effects of dehydration, oral rehydration, and patient risk factors. Am J Roentgenol 207:931-938
32 Yang MS, Choi SI, Song WJ et al (2015) Impact of an electronic consultant system on hypersensitivity reactions to iodinated radiocontrast media: an observational study. Postgrad Med J 91:193-199
33 Vijayalakshmi K, Kunadian B, Wright RA et al (2007) A prospective randomised controlled trial to determine the early and late reactions after the use of iopamidol 340 (Niopam) and iomeprol 350 (Iomeron) in cardiac catheterisation. Eur J Radiol 61:342-350
34 Ho AL, O'Malley ME, Tomlinson GA (2007) Adverse events with universal use of iodixanol for CT: comparison with iohexol. J Comput Assist Tomogr 31:165-168
35 Nagamoto M, Gomi T, Terada H, Terada S, Kohda E (2006) Evaluation of the acute adverse reaction of contrast medium with high and moderate iodine concentration in patients undergoing computed tomography. Radiat Med 24:669-674
36 Wendt-Nordahl G, Rotert H, Trojan L et al (2006) Intravenous contrast media in uroradiology: evaluation of safety and tolerability in almost 50,000 patients. Med Princ Pract 15:358-361
37 Masui T, Katayama M, Kobayashi S, Sakahara H (2005) Intravenous injection of high and medium concentrations of computed tomography contrast media and related heat sensation, local pain, and adverse reactions. J Comput Assist Tomogr 29:704-708
38 Sutton AG, Finn P, Grech ED et al (2001) Early and late reactions after the use of iopamidol 340, ioxaglate 320, and iodixanol 320 in cardiac catheterization. Am Heart J 141:677-683
39 Sutton AG, Finn P, Campbell PG et al (2003) Early and late reactions following the use of iopamidol 340, iomeprol 350 and iodixanol 320 in cardiac catheterization. J Invasive Cardiol 15:133-138
40 Manke C, Marcus C, Page A, Puey J, Batakis O, Fog A (2003) Pain in femoral arteriography. A double-blind, randomized, clinical study comparing safety and efficacy of the iso-osmolar iodixanol 270 mgI/ml and the low-osmolar iomeprol 300 mgI/ml in 9 European centers. Acta Radiol 44:590-596
41 Justesen P, Downes M, Grynne BH, Lang H, Rasch W, Seim E (1997) Injection-associated pain in femoral arteriography: a European multicenter study comparing safety, tolerability, and efficacy of iodixanol and iopromide. Cardiovasc Intervent Radiol 20:251-256
42 Vergara M, Seguel S (1996) Adverse reactions to contrast media in CT: effects of temperature and ionic property. Radiology 199:363-366
43 Tveit K, Dardenne AN, Svihus R et al (1995) Iohexol in patients undergoing urography: a comparison of polypropylene containers (Unique Soft Pack) and glass vials. Clin Radiol 50:44-48
44 McCullough M, Davies P, Richardson R (1989) A large trial of intravenous Conray 325 and Niopam 300 to assess immediate and delayed reactions. Br J Radiol 62:260-265

45 Zeng W, Tang J, Xu X et al (2024) Safety of non-ionic contrast media in CT examinations for out-patients: retrospective multicenter analysis of 473,482 patients. Eur Radiol:1-8
46 McDonald JS, Larson NB, Schmitz JJ et al (2023) Acute Adverse Events After Iodinated Contrast Agent Administration of 359,977 Injections: A Single-Center Retrospective StudyMayo Clinic Proceedings. Elsevier, pp 1820-1830
47 Endrikat J, Michel A, Kölbach R, Lengsfeld P, Vogtländer K (2020) Risk of Hypersensitivity Reactions to Iopromide After Intra-Arterial Versus Intravenous Administration: A Nested Case-Control Analysis of 133,331 Patients. Invest Radiol 55:38-44
48 Gorodetski B, Heine O, Wolf M et al (2020) Safety Analysis of Iobitridol as a Nonionic Contrast Medium: A Postmarketing Multicenter Surveillance Study With 94,960 Patients Almost 20 Years After Introduction. Invest Radiol 55:144-152
49 Chen JY, Liu Y, Zhou YL et al (2015) Safety and tolerability of iopromide in patients undergoing cardiac catheterization: real-world multicenter experience with 17,513 patients from the TRUST trial. Int J Cardiovasc Imaging 31:1281-1291
50 Palkowitsch PK, Bostelmann S, Lengsfeld P (2014) Safety and tolerability of iopromide intravascular use: a pooled analysis of three non-interventional studies in 132,012 patients. Acta Radiol 55:707-714
51 Maurer M, Heine O, Wolf M, Freyhardt P, Schnapauff D, Hamm B (2011) Safety and tolerability of iobitridol in general and in patients with risk factors: results in more than 160,000 patients. Eur J Radiol 80:357-362
52 Callahan MJ, Poznauskis L, Zurakowski D, Taylor GA (2009) Nonionic iodinated intravenous contrast material-related reactions: incidence in large urban children's hospital--retrospective analysis of data in 12,494 patients. Radiology 250:674-681
53 Vogl TJ, Honold E, Wolf M, Mohajeri H, Hammerstingl R (2006) Safety of iobitridol in the general population and at-risk patients. Eur Radiol 16:1288-1297
54 Mortelé KJ, Oliva MR, Ondategui S, Ros PR, Silverman SG (2005) Universal use of nonionic iodinated contrast medium for CT: evaluation of safety in a large urban teaching hospital. Am J Roentgenol 184:31-34
